# Vitamin D Supplementation as a Therapeutic Strategy in Autoimmune Diabetes: Insights and Implications for LADA Management

**DOI:** 10.3390/nu16234072

**Published:** 2024-11-27

**Authors:** Niki G. Mourelatou, Dimitris Kounatidis, Edward B. Jude, Eleni Rebelos

**Affiliations:** 1 Second Department of Internal Medicine, NIMTS Hospital, 11521 Athens, Greece; nikimourelatou@yahoo.gr; 2 Diabetes Center, First Department of Propaedeutic and Internal Medicine, Laiko General Hospital, Medical School, National and Kapodistrian University of Athens, 11527 Athens, Greece; dimitriskounatidis82@outlook.com; 3 Tameside and Glossop Integrated Care NHS Foundation Trust, Ashton-under-Lyne OL6 9RW, UK; 4 Division of Cardiovascular Sciences, Faculty of Biology, Medicine and Health, University of Manchester, Manchester M13 9PL, UK; 5 Faculty of Science & Engineering, Manchester Metropolitan University, Manchester M15 6BX, UK; 6 Department of Clinical and Experimental Medicine, University of Pisa, 56126 Pisa, Italy; eleni.rempelou@unipi.it

**Keywords:** autoimmune diabetes, dipeptidyl peptidase-4 inhibitors, LADA, T1D, vitamin D, vitamin D deficiency

## Abstract

Latent autoimmune diabetes of adults (LADA) is the most prevalent form of autoimmune diabetes (AI-D) in adulthood; however, its accurate diagnosis and optimal treatment remain challenging. Vitamin D deficiency (VDD) is commonly observed in LADA patients, while increased vitamin D exposure through supplementation and dietary intake is associated with a reduced incidence of LADA. Although limited, case reports, case-control studies, and randomized clinical trials have examined the effects of vitamin D supplementation—alone or combined with dipeptidyl peptidase-4 inhibitors (DPP4-is)—on glucose regulation, residual β-cell function, and glutamic acid decarboxylase antibody (GADA65) levels. Findings, while preliminary, indicate that vitamin D supplementation may enhance glycemic control, preserve β-cell function, and reduce autoimmune activity. Given its accessibility, affordability, and relative safety, vitamin D supplementation presents an attractive adjunct treatment option for LADA patients. This narrative review discusses current evidence on the potential therapeutic benefits of vitamin D supplementation in patients with AI-D, including LADA, who are also vitamin D deficient. Beginning with an exploration of the epidemiological patterns, clinical presentation, and diagnostic framework essential for understanding and identifying LADA, this review then examines the proposed mechanisms through which vitamin D may influence autoimmune modulation of pancreatic β-cells, integrating recent data pertinent to LADA pathology. By distilling and consolidating existing research, we aim to provide a platform for advancing targeted investigations within this distinct patient population.

## 1. Introduction

Diabetes mellitus (DM) has emerged as a global pandemic and a significant public health challenge. According to the International Diabetes Federation (IDF), 537 million adults worldwide were living with diabetes in 2021, a number projected to increase to 643 million by 2030 and 783 million by 2045 [1]. Although the majority of this prevalence surge is attributed to type 2 diabetes (T2D), the incidence of type 1 diabetes (T1D) has also seen a substantial rise [2].

T1D is characterized by the autoimmune destruction of pancreatic β-cells, progressing through three distinct stages. In the first stage, β-cell autoimmunity is present, indicated by two or more islet autoantibodies, though glucose tolerance remains normal. In the second stage, dysglycemia develops as glucose regulation begins to decline. Finally, in the third stage, glucose control worsens further, leading to the onset of clinical symptoms and overt DM. While earlier assumptions suggested that approximately 80% of β-cells are destroyed by the time of diagnosis [3,4], more recent evidence indicates that residual endogenous insulin secretion may persist even in patients with long-standing T1D [5]. As β-cell destruction and functional decline continue over time [4], early disease detection and the timely implementation of disease-modifying interventions could play a crucial role in preserving β-cell function.

Latent autoimmune diabetes of adults (LADA) is the most prevalent autoimmune form of diabetes in adulthood, accounting for approximately 2–12% of all people with DM [6]. The term “latent” in LADA reflects that this autoimmune diabetes (AI-D) subtype cannot be diagnosed without testing for diabetes-related autoantibodies [7]. Although there is still debate for its definition, LADA is often regarded as a subtype of T1D [8], though it is more accurately described as a unique form of diabetes that bridges T1D and T2D, exhibiting characteristics of both and frequently referred to as “type 1.5 diabetes” [9]. Currently, the diagnosis of LADA is heavily based on criteria established by the Immunology for Diabetes Society (IDS). These criteria include the later onset of diabetes, typically after the age of 30, the presence of islet autoantibodies, and an initial period of insulin independence lasting at least six months post-diagnosis. While these criteria provide a useful framework, they may lack the definitive nature found in the diagnostic criteria for T1D and T2D, largely due to the fact that the decision to initiate insulin therapy is often influenced by clinical judgment and individual patient circumstances [6,8].

A significant body of preclinical and clinical research suggests a potential role of vitamin D deficiency (VDD) in the pathogenesis of AI-D, with evidence indicating that vitamin D therapy may mitigate AI-D [10,11]. The majority of these data pertain to cases of T1D. Nevertheless, emerging evidence suggests that the immunomodulatory actions of vitamin D may also confer benefits on β-cell function in cases of LADA [12,13].

This narrative review seeks to explore the connection between AI-D and vitamin D, summarizing current insights and highlighting potential implications for patient care and therapeutic approaches, particularly in patients with LADA. Given the limited number of studies focusing exclusively on LADA, we also present data on VDD and vitamin D supplementation in T1D patients as a comparative basis.

## 2. Pathophysiology of LADA

The pathogenesis of LADA reveals overlapping mechanisms with both T1D and T2D, often leading to diagnostic challenges. T1D, although predominantly diagnosed in childhood, can also manifest later in life [14]. It is characterized by progressive autoimmune β-cell destruction, driven by autoantibodies that rapidly result in insulin deficiency, hyperglycemia, and a dependence on exogenous insulin [15]. In contrast, T2D typically develops in older adults [14] and is primarily defined by insulin resistance in key tissues, including the liver, muscle, and adipose tissue. This impaired response to insulin exacerbates hyperglycemia through processes such as increased glycogenolysis and hepatic gluconeogenesis. As insulin resistance intensifies, pancreatic β-cells are pushed to produce more insulin to meet metabolic demands, ultimately leading to β-cell exhaustion and impaired insulin secretion [16]. LADA displays a combination of these characteristics, with varying levels of autoimmune β-cell destruction and insulin resistance [17].

In T1D, autoimmunity involves heightened inflammatory cytokine production and increased activity of T helper (Th)17 lymphocytes, along with reduced regulatory T lymphocyte (Treg) function, an imbalance shaped by both genetic and environmental factors. Pancreatic β-cell destruction in T1D is primarily mediated by cytotoxic T lymphocytes (CD8^+^ T-cells) [18]. The most commonly detected autoantibodies in T1D are glutamic acid decarboxylase 65 antibodies (GADA65); however, insulin autoantibodies (IAAs), insulinoma-associated antigen-2 autoantibodies (IA-2A), islet cell cytoplasmic autoantibodies (ICAs), and zinc transporter-8 autoantibodies (ZnT8A) are also frequently present [19,20]. Notably, an inverse relationship between GADA65 and C-peptide levels has been identified, indicating that autoantibody levels may reflect both the presence and the extent of β-cell destruction [21]. Moreover, the degree of autoimmunity and β-cell failure is closely associated with insulin treatment requirements [21].

Genetically, LADA shares similarities with both T1D and T2D. The primary genetic associations with T1D involve human leukocyte antigen (*HLA*) genes, with an elevated prevalence of the *HLA DR3*, DQB1*0201, and *DR4*, DQB1*0302 alleles [22,23]. These genes encode the major histocompatibility complex (MHC), a crucial immune system regulator [23]. This genetic pattern suggests a link between LADA and T1D, positioning LADA as a continuation of T1D’s autoimmune pathology, albeit with an adult onset [24]. Individuals with LADA typically display lower frequencies of T1D risk alleles compared to those diagnosed with T1D in childhood, whereas the genetic risk profile of those diagnosed after the age of 30 more closely resembles that of adult-onset T1D. T1D risk alleles, such as those in *HLA*, *INS*, and *PTPN22*, appear more frequently in LADA than in T2D, indicating a stronger autoimmune component [25]. In contrast, LADA’s genetic connections with T2D include various genes across the genome, though these associations are less pronounced [23]. Shared risk factors between LADA and T2D, such as being overweight, adiposity, physical inactivity, smoking, and low birth weight, underscore a component of insulin resistance in LADA, albeit to a lesser extent than in T2D [26].

Based on these genetic and environmental influences, a model for LADA development suggests that an underlying autoimmune genetic predisposition initiates gradual β-cell destruction, followed by exposure to lifestyle factors that contribute to insulin resistance. This cumulative effect may lead to hyperglycemia and LADA. Although lifestyle interventions may hold potential for LADA prevention, more research is needed to confirm their effectiveness [27]. A Norwegian genetic study highlights a strong association between T1D *HLA* genes and high-GADA65 LADA patients, while two specific *HLA* haplotypes correlate with low-GADA65 LADA [28]. Additionally, T2D-related genes, such as CC/CT genotypes of rs7961581 (*TSPAN8*) and obesity-linked AA/AC genotypes of rs8050136 (*FTO*), are predominantly associated with low-GADA65 LADA patients [28]. The first genome-wide association study (GWAS) on LADA has corroborated this dual genetic profile of autoimmune T1D and metabolic T2D, further supporting the hypothesis that LADA may result from a “second hit” of T2D metabolic stress on an existing moderate autoimmune susceptibility. Moreover, locus *PFKFB3* has emerged as a potential identifier of LADA, though further investigation is required [29].

## 3. Epidemiology, Clinical Presentation, and Diagnostic Work-Up in LADA

LADA is the most common form of AI-D among adults, accounting for approximately 2–12% of total DM cases in this population [30]. Research indicates that an estimated 5–10% of patients initially diagnosed with T2D are actually misdiagnosed cases of LADA [31]. Beyond genetic predisposition, several lifestyle risk factors, similar to those associated with T2D, appear to contribute to LADA pathogenesis. Smoking, adiposity, being overweight, and low birth weight are recognized as significant risk factors, while regular physical activity has been shown to reduce risk [32,33]. Among dietary habits, a balanced diet emerges as a critical component in lowering LADA risk. High consumption of sweetened beverages and red or processed meats is positively associated with increased risk, whereas fatty fish intake demonstrates a protective effect. Moderate alcohol consumption may also confer some protection, while coffee intake, particularly when interacting with *HLA* genetic factors, has been linked to greater susceptibility to LADA [34,35,36,37].

LADA presents heterogeneously, ranging from diabetic ketoacidosis to mild, non-insulin-dependent DM, with a slower β-cell dysfunction progression compared to T1D [17,38]. Younger cases, termed LADY (latent autoimmune diabetes of the young), exhibit similar patterns [39]. LADA patients often begin with lifestyle modifications and oral hypoglycemics but may require insulin as β-cell function declines. Insulin-dependent LADA is associated with higher GADA65 autoantibodies and lower C-peptide levels [19]. The United Kingdom Prospective Diabetes Study (UKPDS) found most GADA65-positive individuals initially diagnosed with T2D progressed to insulin dependence within six years, with autoantibody levels inversely related to age and correlating with lower body mass index (BMI) and higher hemoglobin A1c (HbA1c) in younger patients [40].

**Figure 1:**
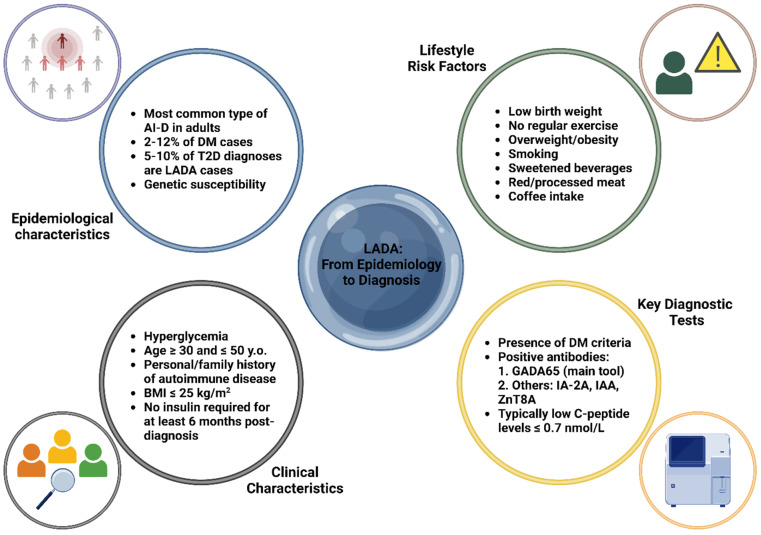
Key epidemiological, clinical, and diagnostic characteristics of latent autoimmune diabetes in adults (LADA) [6,19,30,31,32,33,34,35,36,37,38,39,40,41,42]. Abbreviations: AI-D: autoimmune diabetes; BMI: body mass index; DM: diabetes mellitus; GADA65: glutamic acid decarboxylase antibody 65; IA-2A: islet antigen-2 antibody; IAA: insulin autoantibodies; LADA: latent autoimmune diabetes in adults; T2D: type 2 diabetes; y.o.: years old; ZnT8A: zinc transporter 8 antibody. Created with www.BioRender.com.

LADA patients typically have a lower BMI, reduced insulin resistance, and fewer metabolic syndrome features than T2D, with a potential autoimmune disease history [6]. Diagnosis criteria include age > 30, specific autoantibodies, and delayed insulin dependence (>6 months), though overlap with T1D and T2D complicates classification [6]. Clinical features like age < 50, BMI < 25 kg/m^2^, autoimmune history, and acute symptom onset suggest LADA, warranting GADA65 antibody screening, especially in T2D patients under 60 with low BMI or poor metabolic control [6,41]. C-peptide measurement aids diagnosis: <0.3 nmol/L suggests T1D, >0.7 nmol/L suggests T2D, and 0.3–0.7 nmol/L requires periodic reassessment. Insulin therapy may eventually be necessary, with T2D treatment used cautiously to avoid further β-cell compromise [6,42]. Figure 1 outlines the essential characteristics of LADA, covering aspects of both epidemiological and clinical features, as well as the primary laboratory tools utilized for diagnosis.

## 4. Vitamin D’s Role in Immune Regulation and Pancreatic β-Cell Function: Mechanistic Insights into Autoimmune Diabetes

Vitamin D is a fat-soluble steroid and a precursor to human steroid hormones, originating from two primary natural sources: dietary vitamin D, which includes ergocalciferol (D_2_) and cholecalciferol (D_3_), and vitamin D_3_ generated through exposure to ultraviolet B (UVB) sunlight [43]. Dietary sources provide only about 20% of the recommended daily intake of this nutrient [44]. Both D_3_ and D_2_ forms of vitamin D are biologically inactive until metabolized in the skin, liver, and kidneys. In the skin, 7-dehydrocholesterol is converted into pre-vitamin D_3_, which then forms vitamin D_3_ and subsequently enters the bloodstream. Concurrently, dietary vitamin D_3_ and D_2_ are absorbed from the intestinal lumen into the blood. All circulating vitamin D is transported to the liver, where it is converted into the 25-hydroxyvitamin D (25(OH)D) metabolite. Finally, 25(OH)D travels from the liver to the kidneys, where it is transformed into the biologically active metabolite 1,25-dihydroxyvitamin D_3_ (1,25(OH)_2_D_3_), which binds to the vitamin D receptor (VDR), a steroid hormone-responsive member of the nuclear receptor superfamily [43,45].

### 4.1. Immunomodulatory Mechanisms of Vitamin D in Cellular Immune Regulation and β-Cell Protection

Among its biological effects, vitamin D has shown significant immunomodulatory potential, as substantiated by extensive research. This effect is attributed to the active form of vitamin D, 1,25(OH)_2_D_3_, which downregulates mechanisms linked to adaptive immunity, thus promoting immune tolerance and exerting anti-inflammatory actions [46]. This regulatory capability is closely associated with the presence of VDRs on nearly all immune cells, including antigen-presenting cells (APCs) such as macrophages, dendritic cells (DCs), and T-cells (CD4⁺ and CD8⁺) [47]. Notably, many of these cells express the enzyme 1α-hydroxylase (CYP27B1), enabling them to convert circulating 25(OH)D into its active form [46].

The immunomodulatory effects of vitamin D on macrophages are complex and multidimensional. Vitamin D reduces the production of pro-inflammatory cytokines, including interleukin (IL)-1β, IL-6, and tumor necrosis factor-alpha (TNF-α), while it enhances the production of anti-inflammatory cytokines, particularly IL-10 [46,48]. This cytokine shift promotes a phenotypic change in macrophages from a pro-inflammatory (M1) to an anti-inflammatory (M2) profile [49]. Concurrently, vitamin D increases macrophage phagocytic activity, while attenuating their differentiation, activation, and antigen-presenting function [46,47]. Recent research has highlighted vitamin D’s ability to influence the expression of various genes and microRNAs associated with inflammation and cellular stress, thereby affecting the transcriptional activity of monocyte-derived macrophages and resulting in significant changes that drive inflammatory responses [50].

In DCs, vitamin D induces a tolerogenic phenotype, which mitigates inflammation within the pancreatic β-cell microenvironment. This effect is achieved by decreasing pro-inflammatory cytokines, such as IL-12 and TNF-α, while increasing anti-inflammatory cytokines like IL-10 and transforming growth factor-beta (TGF-β) [51,52]. Furthermore, vitamin D modulates T-cell responses by reducing CD8⁺ T-cell hyperreactivity and facilitating CD4⁺ T-cell differentiation into Th2 and Treg cells, which tend to dominate over pro-inflammatory Th1 and Th17 cells. The decrease in pro-inflammatory cytokine secretion, including interferon-gamma (IFN-γ), IL-17, and IL-22, along with an increase in anti-inflammatory cytokines such as IL-4 and IL-10, creates a supportive environment for β-cell health [52,53]. Notably, vitamin D’s interference with the antigen-presenting capacity of immune cells leads to T-cell anergy, thereby diminishing B-cell proliferation. As a result, B-cells have a reduced likelihood of differentiating into memory B-cells and plasma cells, which translates to lower immunoglobulin and autoantibody production [54,55].

### 4.2. The Role of Vitamin D in Pancreatic β-Cell Function and Insulin Secretion

In addition to its immunomodulatory functions, vitamin D plays a pivotal role in the physiology of pancreatic β-cells. The presence of the enzyme 1α-hydroxylase, essential for converting vitamin D into its active form, along with the VDR and vitamin D-binding protein (DBP), suggests a significant role for vitamin D in β-cell physiology [56]. Vitamin D response elements (VDREs) in the promoter region of the insulin gene further imply that vitamin D may enhance insulin secretion by influencing β-cell functionality and calcium homeostasis, a process critical for insulin release [57,58,59]. The advantageous impact of vitamin D on maintaining β-cell viability and bolstering anti-inflammatory responses has been substantiated in preclinical models. Investigations involving non-obese diabetic (NOD) mice indicate that adequate concentrations of vitamin D correlate with a reduced incidence of T1D. Moreover, oral supplementation with 1,25(OH)_2_D_3_ has demonstrated a remarkable capacity to confer complete protection against insulin-dependent DM within these experimental frameworks [60,61].

**Figure 2:**
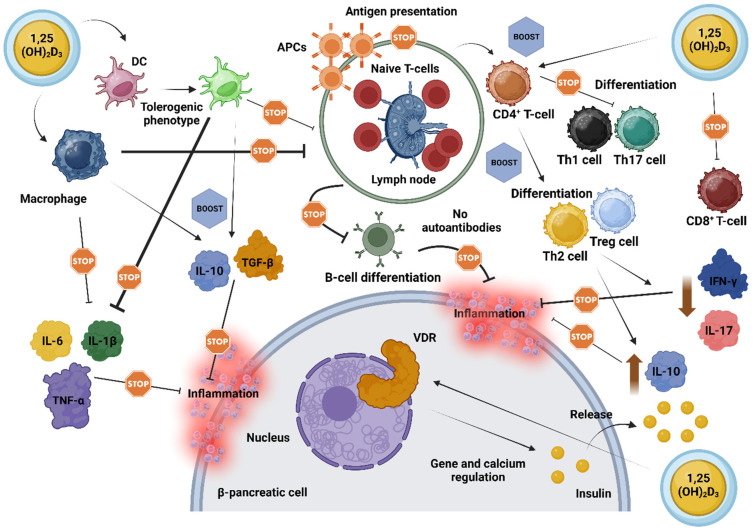
‘Stops and Boosts’: A schematic illustration of the cumulative impact of vitamin D on resilience and functional support of pancreatic β-cells in autoimmune diabetes. Vitamin D, in its active form (1,25(OH)_2_D_3_), exerts broad immunomodulatory effects by engaging its receptors (VDRs) on immune cells, thereby orchestrating a shift towards immune tolerance. This shift involves downregulating pro-inflammatory molecules such as IL-1β, IL-6, and TNF-α, while potentiating anti-inflammatory signals, including IL-10 and TGF-β. In macrophages, vitamin D limits the release of inflammatory cytokines, enhances phagocytosis, and diminishes APC activity, thereby decreasing their role in driving immune activation. DCs, similarly influenced by vitamin D, adopt a phenotype that supports cellular tolerance and a balanced immune environment near β-cells. Additionally, vitamin D directs T-cell responses by moderating CD8^+^ T-cell activity and promoting the differentiation of CD4^+^ T-cells towards anti-inflammatory Th2 and Treg phenotypes, while suppressing pro-inflammatory Th1 and Th17 cells. This effect leads to decreased levels of IFN-γ and IL-17, while fostering a milieu that favors β-cell preservation. By impairing the presentation of antigens to T-cells, vitamin D also diminishes B-cell activation and proliferation, thus lowering the production of potentially harmful autoantibodies. Beyond immune modulation, vitamin D aids pancreatic β-cell function directly by enhancing insulin secretion through VDR-mediated gene expression and calcium homeostasis, essential for optimal insulin release [46,47,48,49,50,51,52,53,54,55,56,57,58,59,60,61,62,63,64,65]. Abbreviations: 1,25(OH)_2_D_3_: 1,25-Dihydroxyvitamin D_3_; APC: antigen-presenting cell; B-cell: B lymphocyte; CD4^+^ T-cell: helper T-cell; CD8^+^ T-cell: cytotoxic T-cell; DC: dendritic cell; IFN-γ: interferon gamma; IL: interleukin; TGF-β: transforming growth factor beta; Th: T helper cell; TNF-α: tumor necrosis factor alpha; Treg: regulatory T-cell; VDR: vitamin D receptor. Created with www.BioRender.com.

Pancreatic cells express the VDR gene and the DBP, and certain polymorphisms in the VDR gene may be linked to glucose intolerance and insulin sensitivity, possibly predisposing individuals to the development of T1D [56,61,62]. Notably, overexpression of VDR in genetically modified rodents has conferred protection against the diabetogenic effects of streptozotocin, a compound commonly used to induce DM [63]. Conversely, rodents lacking a functional VDR exhibited reduced insulin secretion [64]. In this context, vitamin D may be particularly beneficial due to its effects on lymphocyte activity and interleukin production, facilitating the downregulation of pro-inflammatory agents while promoting anti-inflammatory responses. Figure 2 depicts the proposed mechanisms by which vitamin D impacts AI-D, including LADA, emphasizing its vital role in regulating β-cell function, modulating immune responses, and enhancing anti-inflammatory pathways.

## 5. The Potential Immunomodulatory Therapeutic Impact of Vitamin D on Autoimmune Diabetes: Evidence from Clinical Studies

### 5.1. Evidence of Vitamin D Efficacy in Patients with Type 1 Diabetes

Vitamin D’s immunomodulatory properties have prompted significant research into its potential role in managing immune responses in T1D, particularly in preserving β-cell function and slowing disease progression. Evidence suggests that VDD is widespread among individuals with T1D, and vitamin D supplementation may support immune modulation, potentially mitigating β-cell destruction [12,66]. A study by Pozzilli et al. compared vitamin D levels in 88 newly diagnosed T1D patients to 57 healthy controls, finding significantly lower plasma levels of 25(OH)D and 1,25(OH)_2_D_3_ in T1D patients. They proposed that vitamin D_3_ supplementation could support a Th2 immune response, which may protect β-cells from autoimmune attack [67]. Similarly, a meta-analysis by Yang et al. revealed a 45% prevalence of VDD among children and adolescents with T1D, suggesting that low vitamin D status is a consistent finding across populations with T1D [68].

Longitudinal studies have further examined the effects of early vitamin D supplementation on T1D risk. A large-scale Finnish birth-cohort study of 12,055 pregnant women and their children demonstrated that consistent vitamin D supplementation in infancy was associated with a reduced incidence of T1D when adjusted for multiple socioeconomic and anthropometric variables [69]. Additionally, the EURODIAB multicenter study reinforced the potential of early-life vitamin D supplementation in reducing T1D risk later in life [70]. Notably, genetic factors, such as specific polymorphisms in the VDR gene, have been linked to an increased susceptibility to T1D; for example, the *Bsm I* variant is associated with an accelerated onset of the disease [71,72].

Clinical trials have investigated the effects of various forms of vitamin D on β-cell preservation in T1D patients. In a small trial involving high-risk children, calcitriol (0.25 μg/day) normalized specific autoantibodies over a median of six months, suggesting potential immunoprotective effects [73]. Another study with newly diagnosed T1D patients treated with sitagliptin, a dipeptidyl peptidase-4 inhibitor (DDP-4i), and vitamin D3 observed an extended clinical remission, indicating a potential role for combination therapies in prolonging the honeymoon phase [74]. On the contrary, findings from randomized clinical trials (RCTs) have been mixed regarding vitamin D’s effectiveness in preserving β-cell function. For example, Gabbay et al. conducted a trial with 38 newly diagnosed T1D patients in Brazil which demonstrated that daily cholecalciferol (2000 IU) slowed the decline in C-peptide levels, with no adverse effects when used alongside insulin therapy [75]. Similarly, a study in India with T1D patients who received monthly vitamin D combined with insulin therapy over six months observed significantly higher C-peptide levels compared to controls, although HbA1c levels and insulin requirements remained similar between groups [76].

Despite these promising findings, other studies have shown limited efficacy of vitamin D in preserving β-cell function. For instance, Walter and colleagues observed that daily intake of 1,25(OH)_2_D_3_ at 0.25 μg was safe but did not prevent β-cell dysfunction [77]. Additionally, a separate RCT with T1D patients receiving calcitriol (0.25 μg on alternate days) and nicotinamide showed only modest improvements, with no significant impact on baseline or stimulated C-peptide levels and HbA1c after one year [78]. Similarly, a two-year double-blind trial with 34 T1D patients reported no significant differences in C-peptide reduction or insulin needs between the calcitriol and placebo groups [79]. High-dose vitamin D regimens have also been evaluated in a study using 50,000 IU of ergocalciferol in newly diagnosed T1D patients. While this regimen effectively elevated vitamin D levels and reduced TNF-α, it did not yield significant changes in C-peptide levels or insulin requirements, indicating a limited impact on β-cell preservation at this dose [80].

### 5.2. The Impact of Vitamin D Supplementation in Latent Autoimmune Diabetes of Adults

While substantial research has examined the role of vitamin D in T1D, only a few studies have specifically investigated its impact on individuals with LADA. Bozkuş et al. reported the case of a 55-year-old woman, initially diagnosed with T2D but later reclassified as LADA, following isotretinoin treatment for severe acne. VDD was her only abnormal laboratory finding, leading authors to suggest that isotretinoin’s immunomodulatory effects, combined with VDD, may have triggered the autoimmune condition. This case emphasizes the potential preventive importance of maintaining adequate vitamin D levels in AI-D states [81]. Another case report by Rapti et al. provides additional evidence for the potential benefits of combined treatment approaches in LADA management. The report detailed the case of a young male diagnosed with LADA, confirmed through clinical symptoms and the presence of positive GADA65 antibodies, who was also found to have VDD at the time of diagnosis. The patient was prescribed a combination treatment of cholecalciferol and sitagliptin, a DPP-4i generally used in T2D management, but thought to offer immunomodulatory benefits. The authors proposed sitagliptin as a potential alternative to insulin, hypothesizing that it might extend the insulin-free period for LADA patients. Over two years, the patient maintained excellent glycemic control without insulin and experienced a reduction in GADA65 antibodies to normal levels [82].

Additional evidence comes from a Swedish case-control epidemiological study by Löfvenborg et al., which linked weekly fatty fish consumption—rich in omega-3 fatty acids and vitamin D—with a reduced risk of LADA, though no similar effect was observed for T2D. This protective association was attributed to the anti-inflammatory properties of both vitamin D and omega-3 fatty acids. Notably, maternal intake of vitamin D and omega-3s has been associated with reduced T1D risk in offspring, suggesting a possible long-term protective effect that may extend to LADA [35].

Observational data also emphasize the prevalence and potential effects of VDD in LADA. A study by Tsaryk et al. examined vitamin D levels, carbohydrate metabolism, and GADA antibody levels in 90 patients with DM, including subgroups with T1D, T2D, and LADA, alongside a control group. VDD prevalence was highest in T2D (75%), followed by LADA (67%) and T1D (62%), compared to 12% in healthy controls. Within LADA subgroups, VDD was more common in patients with elevated anti-GADA titers (71% in LADA2 vs. 63% in LADA1). Findings revealed a negative correlation between vitamin D levels, anti-GADA titers, and HbA1C, suggesting that low vitamin D levels may exacerbate autoimmunity and impair glucose regulation in LADA [83].

However, conflicting results emerged from an observational study conducted in Mexico, which examined the relationships between vitamin D intake, serum levels, and markers of insulin secretion and resistance, such as C-peptide and estimated glucose disposal rate (eGDR). Among 107 participants, the study identified positive associations between vitamin D intake and C-peptide levels (r = 0.213; *p* = 0.032) across the cohort and in T2D (*p* = 0.042). Yet, no significant relationships were found for eGDR or between vitamin D levels and insulin secretion in LADA. LADA patients displayed lower vitamin D intake, younger age, lower BMI, and poorer metabolic control than T2D patients. These findings suggest that while vitamin D may influence insulin resistance in T2D, its role in LADA remains inconsistent [84].

The GADinLADA pilot study adds further evidence to vitamin D’s potential role in modulating autoimmunity. This study assessed intralymphatic GAD-alum injections alongside vitamin D supplementation in 14 GADA-positive, insulin-independent LADA patients diagnosed within the prior 36 months. All participants received vitamin D to maintain serum levels above 100 nmol/L. Preliminary findings demonstrated that the treatment was safe and feasible, with stable β-cell function and metabolic control observed during a 5-month follow-up. These results indicate that combining GAD-alum therapy with adequate vitamin D levels may help preserve β-cell function, although further evaluation is needed [13].

Randomized control trials have also shed light on the role of vitamin D in LADA. Over a decade ago, Li et al. conducted a study investigating the effects of alfacalcidol on β-cell function in 35 LADA patients. Participants received either insulin alone or insulin plus alfacalcidol (0.5 μg/day) for one year. Those in the combination group maintained stable fasting C-peptide (FCP) and post-glucose challenge C-peptide (PCP) levels, whereas the insulin-only group experienced significant declines in FCP (*p* = 0.006). Subgroup analysis revealed that patients with DM duration under one year experienced the most pronounced benefits, with 70% maintaining or improving FCP levels compared to 22% in the insulin-only group (*p* < 0.01). Importantly, no severe side effects were reported, underscoring alfacalcidol as a safe adjunctive therapy [85]. Zhang et al. further investigated the combined effects of saxagliptin and vitamin D (2000 IU/day) in a cohort of 60 LADA subjects. Patients receiving this combination alongside conventional therapy maintained stable FCP and PCP levels and consistent C-peptide index (CPI) values over one year. In contrast, those on saxagliptin or conventional therapy alone exhibited significant declines in β-cell function. GADA65 antibody levels were also significantly reduced in the combination group, supporting the potential of saxagliptin and vitamin D to preserve β-cell function in LADA [86].

More recently, a multi-center RCT by Yan et al. evaluated saxagliptin and vitamin D in preserving β-cell function in adult-onset T1D, including LADA. While changes in FCP did not reach significance, the combination therapy group exhibited a smaller decline in 2-h C-peptide area under the curve (AUC) compared to conventional therapy (−276 pmol/L vs. −419 pmol/L; *p* = 0.01). This protective effect was most pronounced in patients with elevated anti-GADA concentrations (*p* = 0.001). Both saxagliptin-containing regimens allowed reduced insulin doses while maintaining comparable glycemic control, offering a promising adjunctive approach for preserving β-cell function in adult-onset autoimmune diabetes, including LADA [87]. Table 1 presents evidence from existing clinical studies examining the potential role of vitamin D supplementation in patients with LADA.

## 6. Vitamin D as a Therapeutic Option in Vitamin D Deficiency and LADA: Challenges, Recommendations, and Future Directions

The prevalence of LADA, the most common form of AI-D in adults, is rising alongside other types of DM. While vitamin D has been widely studied in T1D and T2D, research on its effects in LADA remains sparse, partly due to the diagnostic challenges LADA presents [67,88]. LADA’s overlap with T1D and T2D highlights the need for precise, accessible testing, including autoantibody and serum C-peptide assays, to ensure accurate identification. Personalized treatment strategies are vital, balancing insulin therapy with lifestyle interventions, while addressing diverse patient populations and promoting equitable access to care. The cost-effectiveness of advanced tests and treatments also demands careful evaluation to enhance accessibility. Additional RCTs exploring new drugs and immunotherapies are essential to optimize long-term outcomes and improve the evidence base for managing LADA [89].

The current literature indicates that VDD is prevalent among patients with both T1D and LADA [68,83]. Evidence suggests that VDD during intrauterine development or early childhood increases the risk of developing T1D [90], and these findings may have implications for LADA as well. However, studies exploring the effects of vitamin D in T1D have yielded mixed results. While some research supports a positive correlation between vitamin D supplementation and the preservation of β-cell function—shown by higher levels of C-peptide or slower declines in C-peptide over time—other studies have found no significant differences in C-peptide levels, suggesting that vitamin D may not universally prevent β-cell deterioration. For LADA subjects, the number of RCTs examining the effects of vitamin D supplementation is notably limited [85,86,87]. Nevertheless, the available data imply that vitamin D, alone or combined with DPP4is, may enhance β-cell preservation and reduce autoimmunity [74,82,86]. DPP-4, a multifunctional protein expressed on immune cells, modulates cytokines, chemokines, and peptide hormones, thus playing a significant role in immune regulation and inflammatory responses [91]. Beyond their known effects on the incretin system, DPP4is exhibit immunomodulatory properties in pancreatic islet cells, improving β-cell functionality and survival by mitigating cytokine-induced toxicity, apoptosis, and nuclear factor kappa-light-chain-enhancer of activated B-cell (NF-κB) expression, while enhancing insulin secretion [92]. This raises a hypothesis that in VDD patients with LADA, the combined use of DPP4is and vitamin D may represent a promising intervention. This approach appears particularly effective when initiated early in the disease course or in cases with less extensive autoimmune damage, suggesting greater efficacy in LADA compared to T1D [69,70,83,85,87].

Glucagon-like peptide-1 receptor agonists (GLP-1 RAs) have also garnered significant attention for their dual metabolic and immunomodulatory effects, which are attributed to GLP-1 receptor expression on immune cells [93]. Preclinical research highlights their capacity to reduce β-cell apoptosis, stimulate β-cell regeneration, and decelerate disease progression [94]. Clinical evidence corroborates these findings, demonstrating improved β-cell function and reduced HbA1c levels in patients with LADA [95]. The interplay between GLP-1 RAs and VDD remains underexplored. However, emerging data suggest that weight loss, a hallmark effect of GLP-1 RAs, may enhance serum 25(OH)D levels, raising the possibility of synergistic benefits when combined with vitamin D supplementation [96]. This combination could theoretically amplify immunomodulatory effects, offering a novel therapeutic strategy for patients with concurrent LADA and VDD.

Similarly, sodium-glucose co-transporter-2 (SGLT-2) inhibitors, known for their anti-inflammatory and T-cell modulating properties, show promise in autoimmune conditions like LADA [97]. By reducing systemic inflammation and oxidative stress, these agents may also augment the immunoregulatory effects of vitamin D [98], although a direct mechanistic link between SGLT-2 inhibitors and vitamin D metabolism has yet to be established in LADA. Despite their therapeutic potential, both GLP-1 RAs and SGLT-2 inhibitors are associated with an increased risk of diabetic ketoacidosis (DKA) in LADA patients. For GLP-1 RAs, this risk is heightened when used as a substitute for adequate insulin therapy, while SGLT-2 inhibitors pose greater DKA risk in patients with a BMI < 27 kg/m^2^ or low C-peptide levels [6,99,100]. Future research should explore the synergistic potential of these agents with vitamin D supplementation to establish safe and effective therapeutic strategies for managing LADA in the presence of VDD.

Presently, no specific guidelines exist for vitamin D supplementation in AI-D. The most recent guidelines from the Endocrine Society discourage routine testing for 25(OH)D levels, citing the lack of clearly defined thresholds that correspond with specific clinical outcomes. However, these guidelines do recommend vitamin D supplementation for adults with ‘high-risk pre-diabetes’ as a preventive measure against DM progression, with an emphasis on consistent, low-dose administration over intermittent high doses [101]. Based on existing evidence, we advocate for the assessment of 25(OH)D levels in individuals with AI-D and suggest treatment initiation in cases of confirmed VDD. As emphasized by both our research and others, the standardization of 25(OH)D measurements, alongside a comprehensive analysis of the entire vitamin D endocrine pathway [88,102], may offer greater insights into the effects of VDD and vitamin D supplementation on the mechanisms underpinning autoimmunity and subsequent clinical outcomes.

Although vitamin D supplementation is not a definitive treatment, the positive results thus far, combined with its affordability and low toxicity, position it as a viable therapeutic option for newly diagnosed patients with AI-D, including LADA. Large-scale, long-term studies are needed to further assess vitamin D’s potential as a standalone treatment and in combination regimens with anti-diabetic agents in LADA patients.

## Figures and Tables

**Table 1:** Clinical studies on the role of vitamin D supplementation in patients with LADA.

Author, Year	Study’s Desing	Study’s Objective	Population of the Study	Results	Conclusions
Li, 2009, [85]	RCT	Evaluation of whether the addition of 1-alpha(OH)D_3_ to insulin therapy helps preserve β-cell function in patients with LADA	A total of 35 LADA patients divided into two groups (1-year follow-up): - Group 1 (n = 18): insulin - Group 2 (n = 17): insulin + 1-alpha-(OH)D_3_ (0.5 μg/day)	- FCP and PCP levels remained stable in Group 2, whereas FCP levels exhibited a significant ↓ in Group 1 - After 1 year, 70% of patients in Group 2 either maintained or ↑ their FCP levels, in contrast to only 22% of patients in Group 1 - In subjects with DM duration of 1 year or less, Group 2 showed ↑ preservation of β-cell function, indicated by ↑ FCP and PCP levels	Supplementing insulin therapy with 1-alpha(OH)D_3_ may support β-cell function preservation in LADA patients, particularly in those with a shorter DM duration. This intervention has been shown to be well-tolerated, with no notable adverse effects observed.
Löfvenborg, 2014 [35]	Case-control study	Investigation of the relationship between fish consumption, omega-3 FAs, and the risk of developing LADA compared to T2D	- 89 cases of LADA - 462 cases of T2D - 1007 DM-free controls	- Weekly intake of fatty fish (≥1 serving vs. <1 serving) was correlated with a ↓ risk of LADA but not with T2D - Estimated daily intake of n-3 PUFA (≥0.3 g) demonstrated a similar trend, with an odds ratio for LADA of 0.60 (95% CI 0.35–1.03) and for T2D of 1.14 (95% CI 0.79–1.58) - Supplementation with fish oil was linked to a ↓ risk for LADA (OR 0.47, 95% CI 0.19–1.12), but to a ↑ risk for T2D (OR 1.58, 95% CI 1.08–2.31)	Fatty fish consumption may ↓ the risk of LADA, potentially due to the beneficial effects of marine-derived omega-3 FAs
Cardoso-Sánchez, 2015 [84]	Observational study	Evaluation of the association between vitamin D (both intake and serum levels) and insulin production (C-peptide) and insulin release (eGDR) in adults with T2D and LADA	A total of 107 individuals with the following: - Average age 55.3 ± 11.84 y.o.; - DM duration of 13.23 ± 5.96 years.	- Compared to T2D, LADA patients tend to have lower vitamin D intake, lower BMI, poorer metabolic health, and were generally younger. - While vitamin D intake was initially correlated with insulin secretion across all patients, this link was seen only in T2D after adjustments, with no associations found in LADA.	Vitamin D intake but not serum levels may be associated with insulin resistance in T2D, but neither intake nor serum levels are linked to insulin resistance in LADA.
Zhang, 2020 [86]	RCT	Assessment of whether the addition of vitamin D_3_ to DPP-4i therapy can help preserve β-cell function in patients with LADA	A total of 60 LADA patients were divided into 3 groups: 1. Group A (n = 21): conventional treatment (metformin and/or insulin); 2. Group B (n = 20): SAXA + conventional Treatment; 3. Group C (n = 19): SAXA + vitamin D3 (2000 IU/day) + conventional Treatment.	- Over a 1-year period, Group C maintained stable levels of FCP, PCP, and CPI, indicating preservation of β-cell function. - Group B showed a marked ↓ in FCP, while Group A exhibited a significant ↓ in CPI levels; - Group C also demonstrated a significant ↓ in GADA titers compared to baseline, indicating a potential immune-modulating vitamin D_3_ effect.	Adding vitamin D_3_ to DPP-4i therapy may help sustain β-cell function in LADA patients.
Tsaryk, 2021 [83]	Observational study	Investigation of the relationship between vitamin D levels and carbohydrate metabolism across different DM types, focusing on LADA	- 25 healthy controls and 90 DM patients: 1. 26 with T1D; 2. 28 with T2D; 3. 36 with LADA. - LADA patients divided into two subgroups based on GADA levels (LADA1 vs. LADA2)	- VDD is highly prevalent in DM individuals: 1. 62% in T1D; 2. 75% in T2D; 3. 67% in LADA. - VDD rates: 71% in LADA2 vs. 63% in LADA1 - Correlations in LADA patients: Negative correlations between vitamin D and GADA and HbA1c levels, and a positive correlation with C-peptide levels, indicating vitamin D’s potential link to β-cell function and glycemic control in LADA.	Vitamin D may play a role in the progression of LADA, as VDD in these patients is associated with: - ↑ autoimmunity; - ↓ in β-cell function; - worsened disease management/control.
Björklund, 2022 [13]	Open-label feasibility trial	Evaluation of the safety and feasibility of intranodal GAD-alum injections with vitamin D for LADA patients	A total of 14 GADA-positive, insulin-independent LADA subjects, aged 30–70, diagnosed within 36 months received 4 μg GAD-alum injections at days 1, 30, and 60, along with 2000 U/d vitamin D for 4 months if serum levels were <100 nmol/L	Treatment was safe/feasible, with stable β-cell function and metabolic control observed after 5 months	Intra-lymphatic GAD-alum therapy combined with vitamin D may offer a viable approach for managing LADA, supporting disease stabilization and β-cell preservation.
Yan, 2023 [87]	RCT	Assessment of the effectiveness of SAXA alone and in combination with vitamin D in maintaining β-cell function in LADA patients	A total of 301 DM adults randomly assigned to three groups: - conventional therapy (metformin and/or insulin) (n = 99); - SAXA (n = 100); - SAXA + vitamin D (n = 102).	- The primary outcome of FCP change was not met in SAXA + vitamin D (*p* = 0.18) or SAXA groups (*p* = 0.26); - SAXA + vitamin D showed significantly less ↓ in 2-h C-peptide AUC compared to conventional therapy (−276 pmol/L vs. −419 pmol/L; *p* = 0.01); - SAXA + vitamin D was especially effective in individuals with ↑ GADA levels (*p* = 0.001); - insulin dosage was ↓ in both SAXA groups while maintaining similar glycemic control across groups.	- The combination of SAXA and vitamin D appears to sustain β-cell function in LADA patients, with significant benefits in those with ↑ GADA levels. - This treatment shows potential as an adjunct to conventional therapy, offering a promising early intervention option for LADA subjects.

Abbreviations: AUC: area under the curve; BMI: body mass index; CI: confidence interval; DM: diabetes mellitus; DPP-4i: dipeptidyl peptidase-4 inhibitor; FCP: fasting C-peptide; GADA: glutamic acid decarboxylase autoantibodies; HbA1c: hemoglobin A1c; LADA: latent autoimmune diabetes of adults; n-3 PUFA: omega-3 polyunsaturated fatty acids; PCP: postprandial C-peptide; RCT: randomized controlled trial; SAXA: saxagliptin; T1D: type 1 diabetes; T2D: type 2 diabetes; VDD: vitamin D deficiency; Vitamin D_3_: cholecalciferol. ↑: increase; ↓: decrease.

## Abbreviations

1,25(OH)_2_D_3_: 1,25-Dihydroxyvitamin D3; 25(OH)D: 25-Hydroxyvitamin D; AI-D: autoimmune diabetes; AUC: area under the curve; B-cells: B Lymphocytes; BMI: body mass index; APCs: antigen-presenting cells; CD4⁺: helper T-cells; CD8^+^: cytotoxic T-cells; CI: confidence interval; CPI: C-peptide index; CV: cardiovascular; CYP27B1: 1α-hydroxylase; DCs: dendritic cells; D_2_: ergocalciferol; D_3_: cholecalciferol; DDP-4 i: dipeptidyl peptidase-4 inhibitors; DM: diabetes mellitus; FCP: fasting C-peptide; GADA65: glutamic acid decarboxylase 65 antibodies; GLP-1 RA: glucagon-like peptide-1 receptor agonist; GWAS: genome-wide association study; HbA1c: hemoglobin A1c; *HLA*: human leukocyte antigen; IA-2A: insulinoma-associated antigen-2 autoantibodies; IAA: insulin autoantibodies; ICA: islet cell cytoplasmic autoantibodies; IDF: International Diabetes Federation; IDS: Immunology for Diabetes Society; IFN-γ: interferon-gamma IL: interleukin; INS: insulin gene; LADA: latent autoimmune diabetes in adults; MHC: major histocompatibility complex; NF-κB: nuclear factor kappa-light-chain-enhancer of activated B-cells; n-3 PUFA: omega-3 polyunsaturated fatty acids; NOD: non-obese diabetic; PCP: postprandial C-peptide; RCT: randomized controlled trial; SAXA: saxagliptin; T1D: type 1 diabetes; SGLT-2: sodium-glucose co-transporter-2; T2D: type 2 diabetes; TGF-β: transforming growth factor-beta; Th: T helper cells; TNF-α: tumor necrosis factor-alpha; Treg: regulatory T-cell; UKPDS: The United Kingdom Prospective Diabetes Study; UVB: ultraviolet B; VDD: vitamin D deficiency; VDR: vitamin D receptor; VDREs: vitamin D response elements; y.o.: years old; ZnT8A: zinc transporter-8 autoantibodies.
